# 

**Published:** 1871-12

**Authors:** 


### Contents of December Number.

### ORIGINAL DEPARTMENT.

ART. 1. The Intraocular Serous Membrane and the Nature of Glaucoma. By Dr. A. Sichel, Jr. Translated by F W. Abbott, M. D ... 161
ART. 2. Apyretic Malarial Menorrhagia. By J. W Southworth. M. D ... 174
ART. 3 Clinical Lecture upon Surgical Cases in the Buffalo Hospital of the Sisters of Charity. By Prof. J. F. Miner Reported by Louis Brecht member of the class... 177

### MISCELLANEOUS.

Albany Hospital ... 182
Intemperance in use of Quinine ... 187
Medical Colleges in Chicago ... 187
To the Medical Profession of the United States ... 189
Relief for Medical Men ... 191

### EDITORIAL DEPARTMENT.

The Cuban Murder of Medical Students ... 192
Medical Colleges and Graduates in the United States ... 193

BOOKS REVIEWED:

Bright on Cancer, its Classification and Remedies ... 193
Thoughts on Chronic Inversion of the Uterus. By H. Miller, M. D ... 194
Remarks on Wine and Alcohol. By Charles A. Lee, M D ... 195
The Prevention of Abscesses in Hypodermic Medication. By Reuben A. Vance. M. D ... 195
[illegible]linical Examination of Urine. By Reuben A. Vance. M. D ... 195
A Contribution to the Treatment of Versions and Flexions of the Unimpregnated Uterus. By Ephraim Cutter, M. D ... 195
The Physicians' Dose and Symptom Book. By Joseph Wythes, M. D ... 196
Gems of Pen Arts. By Charles B. Knowlton ... 196
The Scientific American ... 196
Skin Diseases, their Description, Pathology, Diagnosis and Treatment. By Tilbury Fox, M. D. Edited by M. H. Henry, M. D ... 196
Manual of Midwifery. By Alfred Meadows, M. D ... 197
The Clinical Thermometer. By Z C McElroy, M D ... 197
Physicians' Visiting List for 1872 By Lindsay & Blakiston ... 197
Physicians' Daily Pocket Record. By S W Butler. M D ... 197
Report to the Surgeon General on an Improved Method of Photographing Histological Preparations by Sunlight. By Assistant Surgeon J J. Woodward, U. S. A ... 198
American Practitioner ... 198

Wanted ... 198
The Chicago Medical Examiner ... 199
Bowdoin Medical College ... 199
Annual Report of the Surgeon General U S A ... 199
Books and Pamphlets Received ... 200

THE BUFFALO

